# Elucidating the Role of Aqueous Solvent and Cations in Selectivity of Cobalt–Porphyrin Catalyzed CO_2_ Reduction Over Hydrogen Evolution

**DOI:** 10.1002/cphc.202500727

**Published:** 2026-08-12

**Authors:** Lingshu Zhuo, Evert Jan Meijer

**Affiliations:** ^1^ Van ’t Hoff Institute for Molecular Sciences University of Amsterdam Amsterdam The Netherlands

**Keywords:** cation effect, CO_2_ reduction, density functional theory (DFT), hydrogen evolution, selectivity

## Abstract

Density functional theory‐based molecular dynamics simulations with explicit solvent representation were employed to investigate the mechanism of electrochemical reduction of CO_2_‐to‐CO (CO_2_RR), catalyzed by a cobalt porphyrin complex (CoTPP) in aqueous solution. Our study focuses on the role of solvent molecules and a solvated potassium cation. The simulations reveal that solvent molecules play an active role in key reaction steps, significantly lowering the reaction barrier compared to previous results, primarily due to H‐bond stabilization of the transition state. Additionally, the calculations indicate that the presence of water molecules disfavors the competing hydrogen evolution reaction. These findings underscore the importance of incorporating an explicit molecular description of the solvent to accurately estimate reaction energies, providing a realistic model that highlights the high selectivity of the CoTPP catalyst.

## Graphical abstract

Explicit‐solvent simulations uncover how water and cations may actively affect key reaction steps in electrochemical CO_2_ reduction by a CoTPP catalyst. Hydrogen bonding reshapes the reaction energy landscape, suppressing the competing HER. This reveals how solvent plays a role in CoTPP’s preference for CO formation, offering fresh perspectives on catalyst design.

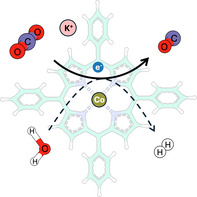

## 1 Introduction

Transforming renewable energy sources such as wind and solar power into more stable forms of energy intended for long‐term storage for later use is one of the great challenges in our modern society. Methods of storing energy in chemical substances are currently receiving attention, especially in the context of the need to convert excess CO_2_ emitted by industry and agriculture. Of the various products of electrochemical CO_2_ reduction (CO_2_RR), CO is a compound that can act as an important chemical building block for valuable products like methanol and methane. However, in addition to electrochemical CO_2_‐to‐CO conversion the reduction of H_2_O to H_2_, via a hydrogen evolution reaction (HER), is a commonly accessible alternative conversion pathway that requires similar overpotentials [1, 2, 3, 4]. This constitutes a crucial challenge and requires a dedicated design of the catalytic system in order to achieve a high selectivity for CO as the main product.

Chemical systems incorporating solvated molecular catalysts can offer an ideal template to reach high activity and selectivity of a catalyzed chemical conversion by structural modifications of the catalyst and well‐tuned composition of the electrolyte solvent. Up to now, extensive research has explored metal porphyrins (MP) for catalyzing CO_2_ reduction [5, 6, 7, 8, 9, 10]. The well‐defined M‐N_4_ macrocyclic structure in MPs serves as the active site and an ideal model for investigating reaction mechanisms in diverse electrocatalytic systems [5, 10, 11]. By modifying the nitrogen‐based macrocyclic porphyrin ligand and altering the central metal atom, the coordination environment and electronic structure of MPs can be tuned at the atomic scale to enhance the targeted catalytic performance. Among the various transition metals, porphyrin complexes based on cobalt achieve a high catalytic performance and will be addressed in this study.

**SCHEME 1:**
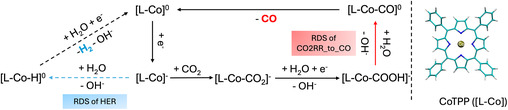
Left: mechanism for CO_2_RR and HER catalyzed by cobalt porphyrin‐based catalyst; right: structure of catalyst CoTPP used in this study (Co: tan, N: dark blue, C: cyan, O: red, and H: white).

To enhance the catalytic performance of the conversion to CO, it is important to understand how MPs are catalytically active and selective for both CO_2_RR and the competing HER [8, 12, 13]. Scheme 1left summarizes the mechanism of CO_2_RR and HER mediated by cobalt‐based porphyrin catalysts in neutral/alkaline media. For the reduction of CO_2_ to CO, the first step constitutes an electron transfer (ET) to the catalyst ([L–Co]^0^ → [L–Co]^−^), followed by the binding of CO_2_ to the cobalt center of the catalyst, forming a Co─C bond. Subsequently, the combination of a protonation step and a one‐electron reduction yields a [L–Co–COOH]^−^ species. The proton‐assisted cleavage of the C─O bond in [L–Co–COOH]^−^, which is the generally considered the rate‐determining step (RDS), yields the desired CO species, still coordinated to the catalyst. The competing HER proceeds along a similar scheme with an initial one‐ET to the Co complex followed by the formation of Co‐hydride, [L–Co–H]^0^. This formation step is considered to be the RDS. The crucial Co‐hydride intermediate yields H_2_ through a proton‐hydride coupling step. A general strategy for enhancing CO production is to tune the catalysts and electrolyte not only to favor the CO_2_RR but also to suppress the HER.

In experimental studies and in practical applications the employed electrolytes have a large range of water concentrations, ranging from almost pure water to systems where there are only traces of H_2_O present [14, 15, 16]. Earlier computational studies addressing CO_2_ conversion using cobalt based porphyrin catalysts either focused on comparing the selectivity of the competing HER and CO_2_RR processes using implicit solvent models [4], or employed explicit water solvent descriptions solely for carbon dioxide reduction reactions [17, 18]. Cations in the electrolyte have been shown to not only maintain electroneutrality but also to influence the electrocatalytic conversion in several interrelated mechanisms [14, 19, 20]. To our knowledge, the role of cations in these processes has not yet been addressed before in a manner that accounts properly for presence of the aqueous solvent and thermal motion.

This paper reports a computational study of reaction pathways and energetics of Co‐porphyrin catalyzed CO_2_RR and HER in aqueous solutions, with a focus on elucidating role of solvent water molecules while also addressing the effect of the presence of cations (Scheme 1right). An accurate atomistic theoretical model incorporating both the electronic structure, molecular dynamics, and an explicit atomistic description of the solvent is employed. Among others, we have shown earlier that accounting for changes in chemical bonding, as well as including thermal motion is essential for properly quantifying reaction paths and the role of solvent water molecules and ions [21, 22, 23, 24].

## 2 Computational Methods

Density functional theory (DFT) molecular dynamics calculations were performed of an atomistic model of CO_2_ conversion catalyzed by CoTPP (simplified as [L–Co] in this article) in aqueous solution, in the presence of a potassium cation. A limited number of static DFT calculations have also been carried out. For the density functional theory‐molecular dynamics (DFT‐MD)simulations, we employed DFT using the Perdew–Burke–Ernzerhof (PBE) exchange–correlation functionals [25], supplemented by D3 dispersion corrections [26]. The simulations were carried out with the CP2K program [27]. Goedecker–Teter–Hutter (GTH) type pseudopotentials are used [25]. A split valence Gaussian basis set designed specifically for these pseudopotentials of double‐ζ quality and with polarization functions (DZVP) was used for all atoms [28]. The auxiliary plane wave basis expansion was cutoff at 400 Ry. Spin unrestricted DFT was used to describe the electronic double/quartet state species.

To model the solvated system we considered the CoTPP–CO_2_ catalyst embedded by 136 water molecules and a single potassium cation. All molecules were placed in a periodically replicated cubic box with an edge length of 17 Å. The size of the box was sufficient to have the reacting region and pottasium cation surrounded by at least two water solvation layers, providing a reasonably proper representation of bulk solvation.

DFT‐MD was carried with a time step of 0.75 fs in the canonical (NVT) ensemble. To control the temperature, the canonical sampling through velocity rescaling thermostat with a coupling constant of 50 fs was used [29]. The DFT‐MD simulations were performed at *T* = 350 K, that yields a closer representation of the behavior of aqueous system at room temperature. It has been observed that DFT based simulations with commonly used GGA functionals somewhat overstructure the water solvent and underestimate the self‐diffusion at ambient temperature [30].

We used the constrained molecular dynamics (cDFT‐MD) method to determine free energy profiles [31, 32]. In this method, using an appropriately chosen reaction coordinate, *Q*, simulations are performed at several fixed values of this coordinate, ranging from *Q*_1_ to *Q*_2_. The free‐energy difference (*Δ* *G*) upon changing from *Q*_1_ to *Q*_2_ is computed using the Equation (1). Here, *F* is the average constraint force measured for each value of *Q* during the constraint DFT‐MD simulation. A more detailed explanation can be found in [32]. For each value of *Q*, a 0.75 ps equilibration run, followed by a 2.25 ps production run, was performed. The data from the production runs was used to compute the average forces and the resulting free energies.

(1) $$\Delta G=-\int_{Q_{1}}^{Q_{2}}\langle F(Q)\rangle \;dQ$$

## 3 Results and Discussion

### 3.1 CO_2_ Reduction Reaction

The coordination of CO_2_ to [L–Co] involves a one‐ET to form complex [L–Co]^−^. This is accompanied by a transformation of [L–Co]^−^ to [L–Co–CO_2_]^−^ In a subsequent step, [L–Co–CO_2_]^−^ will be reduced by a second ET which is accompanied by protonation of the Co‐bonded CO moiety to generate [L–Co–COOH]^−^. This appears to proceed with a with a relatively low barrier (<3 kcal mol^−1^) [4, 33]. The subsequent C─O bond breaking yielding [L–Co–CO]^−^ species has been proposed as the RDS for the electrochemical CO_2_RR when catalyzed by a cobalt porphyrin complex [2, 4, 33].

#### 3.1.1 CO_2_ Binding: Role of Solvent and Cation

Using DFT‐MD simulations, we studied the Co–CO_2_ binding step to assess the explicit effect of the presence of water molecules while also addressing the role of cations, with potassium considered as an example case. Employing the method of constraints (cDFT‐MD), with the cobalt–carbon distance atom as controlled reaction coordinate, the free energy profile was traced for both the singlet and triplet spin state.

**FIGURE 1:**
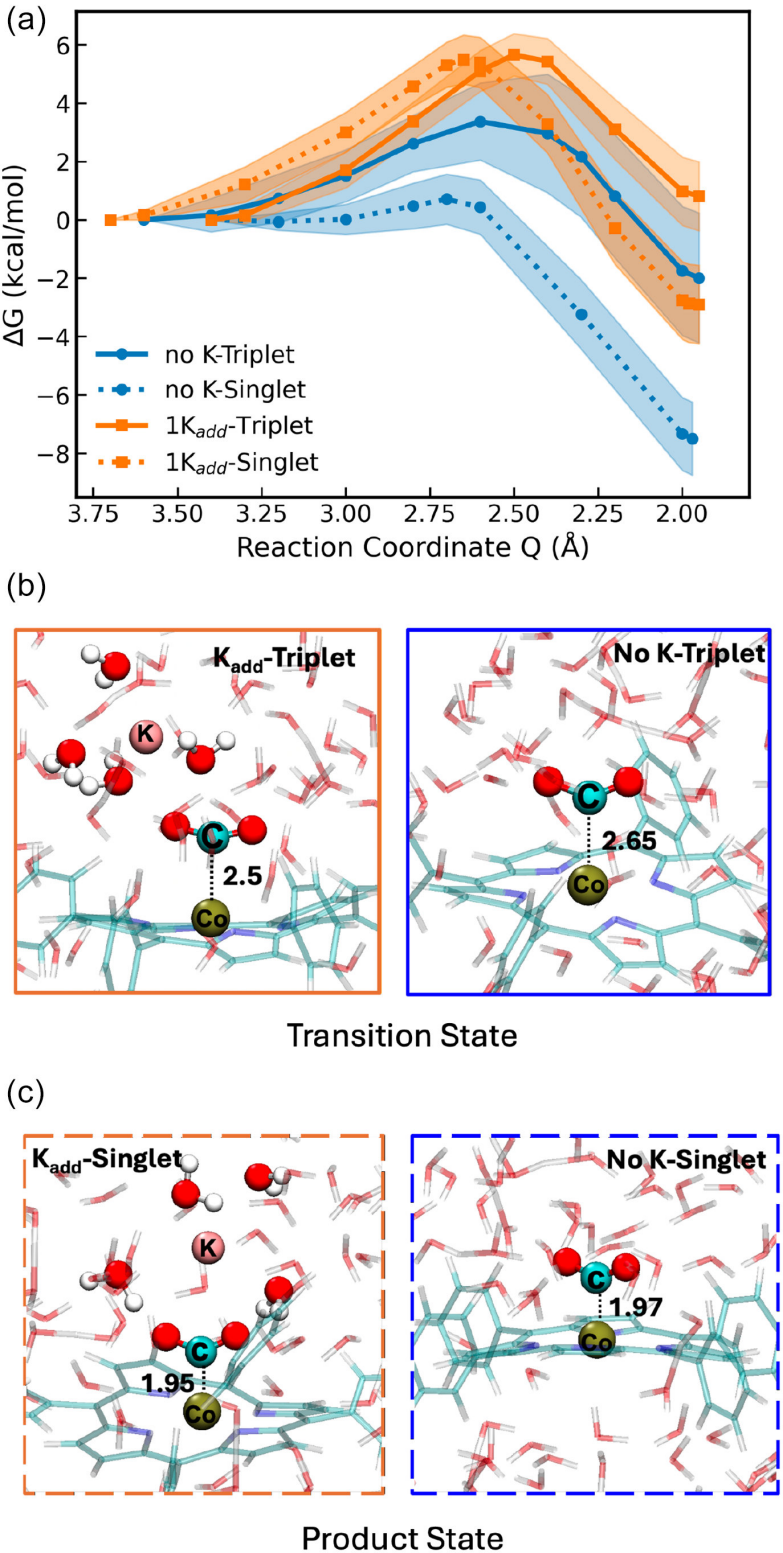
(a) Computed free energy profiles with error bar for the CO_2_ binding without or with K (orange) in CoTPP (singlet in dashed; triplet in solid line) using constrained molecular dynamics with the chosen reaction coordinate (*Q*); (b,c) representative snapshots from the constrained dynamics simulations of transition states and product states respectively.

Figure 1 shows the calculated free energy profiles, together with representative MD‐trajectory snapshots of important stages along the reaction pathway. The current setup does not allow for a reliable estimate of the location of the spin‐state crossing along the reaction path, as we have not determined the relative stability of both spin states in either the reactant or product state. The plotted free energy profiles of the singlet and triplet states should therefore be considered independently. In a pure aqueous solvent the binding proceeds with a negligible of low barrier of 3.0 kcal mol^−1^ for singlet and triplet states, respectively. The CO_2_ binding energy is substantially larger for the singlet spin state, which is consistent with its reported higher stability [4] relative to the triplet spin state.

The presence of nearby cations is expected to play a significant role in the ET steps due to (in)direct electrostatic stabilization. However, the effect on the energetics and mechanism of nonredox stages of the reaction pathway is not well established for homogeneously solvated CO_2_RR catalysts. With a solvated potassium ion nearby the CO_2_ moiety, we traced the CO_2_ binding pathway in the presence of a potassium ion position using cDFT‐MD, which is plotted in Figure 1. Noteworthy, the binding becomes slightly hindered in the presence of a K^+^ cation, with an increase of 2–4 kcal mol^−1^ in free‐energy barrier. This is accompanied by a decrease in the computed binding free energy. Structurally, as shown in Figure 1b, the presence of a potassium cation somewhat shortens the distance between CO_2_ and the cobalt center in the transition state (2.5 Å in *K*_add_‐triplet vs. 2.65 Å in No K‐triplet). The latter indicates an enhanced stability of the reactant state in presence of a potassium cation, which is consistent with the relative decrease of the binding energy. We speculate that this aspect might originate from the change in the solvation structure and associated energetics when proceeding along the reaction path. In the reactant state, potassium is nearly fully water‐coordinated, with CO_2_ also weakly associated (not shown), whereas in the product state of the traced reaction path, the Co‐bonded CO_2_ (a carboxyl‐like moiety) has become part of the first potassium coordination shell (Figure 1c left panel). This change might be less favorable, yielding effectively a suppressing of the binding energy, as observed in the calculated free energy profiles. This argument would imply that a full dissociation of the potassium ion from yields a further decrease in free energy. Assessing and quantifying this step is outside the scope of the current report.

#### 3.1.2 C─O Bond Breaking

**FIGURE 2:**
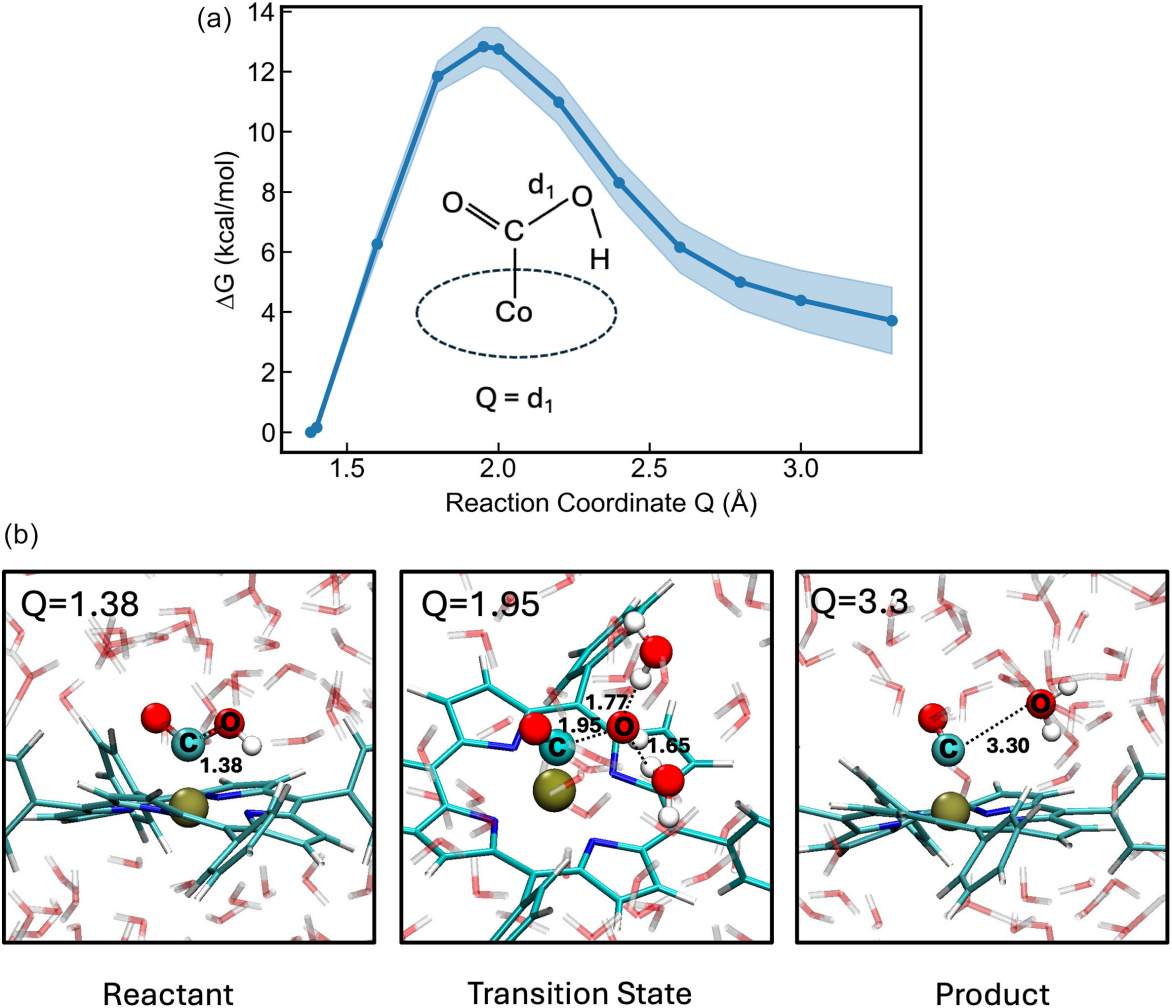
(a) Computed free energy profile with error bar for the C–O cleavage of CoTPP catalysts using constrained molecular dynamics with the chosen reaction coordinate (*Q*) shown inset and (b) representative snapshots from trajectory of reactant, transition state and product state (distances in Å).

Following CO_2_ binding, protonation and a second ET yields the [L–Co–COOH]^−^ intermediate. This step appears to proceeds easily [4], and is not assessed in this study. The subsequent stage involves C─O bond breaking. This will convert the well solvated CoTPP bound CO_2_^−^ moiety to a CoTPP bound CO moiety and a (well solvated) OH^−^ ion. For all proposed mechanisms, this step involves a proton donor moiety protonating the leaving OH group leaving [L–Co–COOH]^−^ complex. In our model system, a solvent water serves as the proton source. To quantify the C─O bond breaking process, we used the method of constraint to vary the C─O distance (*Q*) in a controlled manner. This biased scheme is illustrated in Figure 2a. We determined the free energy profile of for both a pure aqueous system as well for two systems with a potassium cation present. For the latter we considered (1) a system with adding one cation, referred to as K‐add and (2) a system with adding a cation and deprotonating one water molecule, referred to as K‐rpl. This effectively mimics systems with a lower and a higher pH. This setup provides a rather approximate assessment of the effect of varying pH, as and does not properly represents a constant pH condition.

For the pure aqueous system, the calculated free energy profile of the C─O breaking step is shown in Figure 2. It shows reaction energy that is endergonic by ≈4.0 kcal mol^−1^, with a barrier of ≈13.0 kcal mol^−1^. Analysis of the cDFT‐MD trajectories show that the C─O bond breaking is accompanied by a concerted protonation of the leaving OH^−^ group, with the proton originating from surrounding water molecules. This results in the release of a nearby H_2_O molecule, with an OH^−^ group diffusing into the solvent bulk up to a distance of approximately 7 Å from the [L–Co–CO] center. Analysis of the cDFT‐MD trajectory shows that during the concerted C─O breaking, oxygen in the C(O)**O**H moiety forms strong H‐bonds with the protic solvent molecules in the transition state (*Q* = 1.95 Å), as illustrated in Figure 2b, indicating a stabilizing effect. These observations suggests that the C─O breaking pathway involves explicitly solvating water molecules, indicating that accounting for the presence of water molecules is an essential aspect of realistically modeling this reactions step. The product compound [L–Co–CO] does, in the hydrophobic CO region, not form hydrogen bonds with surrounding water molecules.

With a potassium cation present in solution, the DFT‐MD simulations of the reactant state show that the ion coordinates to the COOH moiety in a manner as shown in Figure 1c (left panel). This holds for both the K‐add and K‐rpl system.

The association of the K^+^ cation with the COOH moiety is driven by the overall negative charge to the reactant compound [L–Co–COOH], and the fact that the slightly negatively charged oxygen in the CO group of a COOH group is attractive to cations and hydrogen bond donors. The calculated free energy profiles show that the reaction free‐energy profile, when compared to a pure aqueous system, decreases either negligibly (K‐rpl) or by a small amount (K‐add) with presence of a cation.

**FIGURE 3:**
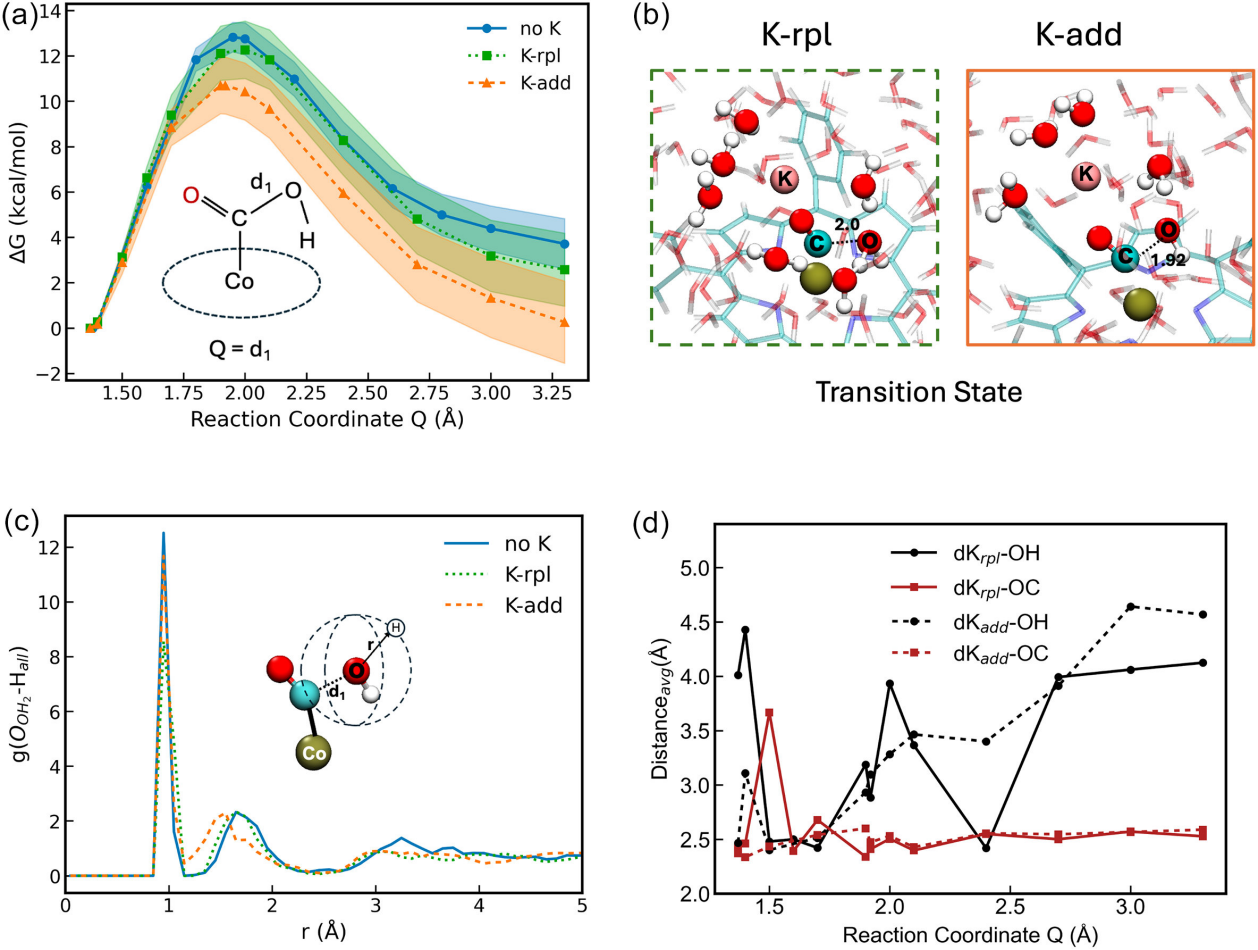
(a) Computed free energy profiles with error bar for the C–O cleavage without (blue) or with K (one K ion replacing a proton in green dotted line; one extra K ion added in orange dashed line) ion in CoTPP using constrained molecular dynamics with the chosen reaction coordinate (*Q*) shown inset; (b) representative snapshots from trajectory of transition state, showing different cation position near the Co–COOH intermediate (K‐rpl: K‐replace‐H_water_ and K‐add: K‐add‐extra respectively); (c) RDF(g) of O_OHC(O)–Co_–H_all_ (illustrated inset) for TPP with K‐rpl and K‐add systems; and (d) the average distances of K atom and **O**C(OH)–Co (dark orange), K ion and OC(**O**H)–Co (black) in systems containing K cation (solid line: K‐rpl; dashed line: K‐add).

To rationalize this observation, we analyzed cDFT‐MD trajectories along the reaction pathway, focusing on the local structure. Tracing the potassium position along the reaction coordinate (Figure 3b,d) shows that it dominantly coordinates with the bare oxygen (dark orange) of the COOH moiety, positioning it in the second (or beyond) coordination shell of the leaving OH^−^ group. This qualitatively explains the absence of a significant effect of the presence of a potassium cation on the free energy profile of C─O bond breaking reaction.

An important aspect of the reaction is hydrogen bonding (before and near the transition state) of the leaving OH group. The strength of this interaction can be assessed by the radial distribution function that measures hydrogen coordination of the OH oxygen. The second peak in the RDFs (O_OHC(O)–Co_–H_all_) reflects hydrogen bonding, corresponding to the distance between the O atom in C(O)**O**H and nearby water hydrogen atoms. Figure 3c shows the RDFs for the transition state cDFT‐MD trajectories. For the pure aqueous and K‐rpl systems, the position (2.0 Å) and shape of the 2nd peak is similar, whereas for the K‐add system, it is shifted to a smaller value (1.92 Å), indicating an enhanced hydrogen bonding. The latter effect might qualitatively rationalize the observed decrease in reaction barrier. Noteworthy, both systems with K^+^ ions promote this C─O breaking in terms of reaction energy (K‐rpl: ≈2 vs. K‐add: ≈0 vs. pure aqueous: ≈4 kcal mol^−1^).

The small enhanced stabilization of the reactant state when comparing the K‐rpl with the K‐add system is near signficant. The extra OH^−^ ion present in the K‐rpl system is always positioned in the second solvation shell (or beyond) of the reaction region, that is, the region with K^+^ or proton‐donating water molecule. Compared to the K‐add system. It therefore appears to play small role from the structural point of view, consistent with the abovementioned small difference between the K‐rpl and K‐add system. Still, the small decrease in stability of the K‐rpl reactant could be argued as being qualitatively consistent, as the presence of an extra OH^−^ in the second solvation shell neighborhood should still yield slightly disfavor the formation of (reactant state) released OH^−^.

A CO moiety is known to bear a small dipole moment with a magnitude and direction that can depend on the chemical environment. For reference, an isolated CO has a dipole moment of around 0.1 D with O being positive. Recent studies indicated that when comparing isolated CO to being bonded in metal–carbonyl complexes, the dipole direction can be in both directions, with dipole values ranging from 0.25 (negative O) to 0.6 (positive O) [34, 35]. Theses studies showed that the dipole magnitude and direction appeared to be correlated with the CO bond length, with bond lengths exceeding that of the isolated CO yielding an effectively more negative O. The DFT‐MD simulations of the free CoTPP–CO systems, both with and without the K cations, show an average CO bond length of 1.16 Å, which is slightly longer than that of the 1.14 Å calculated for the isolated CO. This suggests, in the context of above‐mentioned, that CO oxygen bears a small (possibly positive) effective charge, giving the CO a small dipole moment of up to 0.2 D [34, 35]. In bulk water, the dipole moment of water molecules is larger than 2 D [36, 37]. This suggest that the (electrostatic) interaction of the K cation is with the CO moiety in CoTPP–CO is small, in particular when compared to the interaction with water molecules. This is demonstrated by the observation that in the free DFT‐MD simulation of the K‐rpl system, in the (CoTPP–CO) reactant state shows the K^+^ ion moving slowly away from the CO group, getting solvated by water molecules. As the process of ion diffusion is slow on the time scale accessible for DFT‐MD simulation, a more quantitative analysis of this part of the reaction is currently not feasible.

### 3.2 HER

Metal hydride formation is regarded as the initialization step in the typical proposed pathways for cobalt porphyrin‐catalyzed hydrogen evolution. In homogeneous electrocatalysis, this occurs via proton transfer of a singly reduced [L–Co]^−^ complex by a protic solvent molecule. The generated key intermediate [L–Co–H]^0^ undergoes a second reduction and becomes reactive to couple with a proton from a water molecule, thus producing H_2_.

#### 3.2.1 Metal Hydride Formation

**FIGURE 4:**
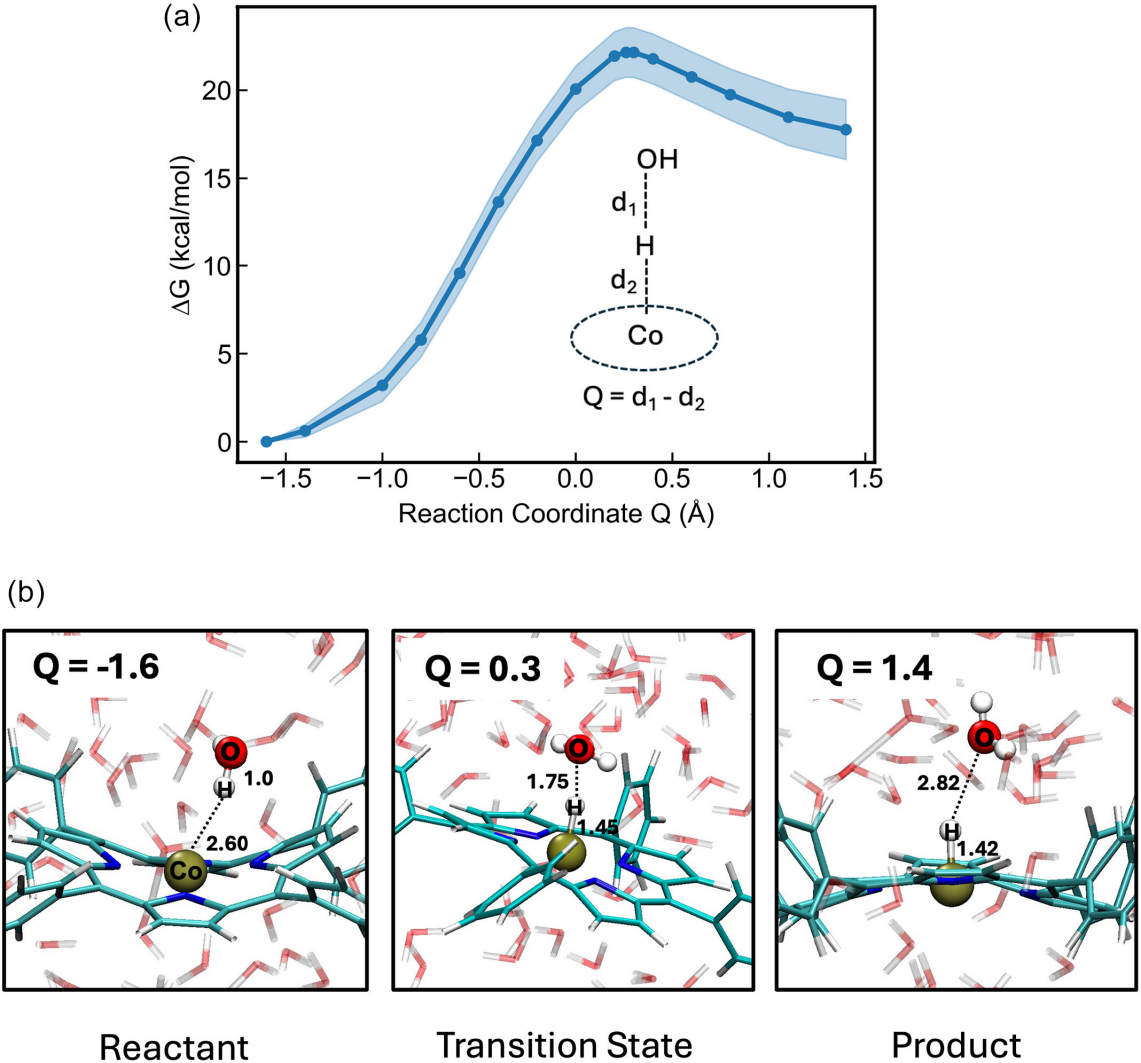
(a) Computed free energy profile with error bar for the singly reduced cobalt hydride formation of CoTPP catalysts using constrained molecular dynamics with the chosen reaction coordinate (*Q*) shown inset and (b) representative snapshots from trajectory of reactant, transition state and product state.

We determined the free energy profile of the initiating proton transfer process using cDFT‐MD. As shown in the inset of Figure 4, the chosen reaction coordinate (*Q*) involves the deprotonation of a water molecule and the simultaneous formation of a Co─H bond. The value of *Q* was varied from −1.6 to 1.4 Å. The calculated free energy profile (Figure 4) shows a barrier of 22.1 kcal mol^−1^, with the reaction energy going uphill by 17.8 kcal mol^−1^. The reaction proceeds in a concerted manner, with proton transfer to Co is accompanied by protonation of the proton‐donating water molecule by surrounding water molecules. The resulting aqueous OH^−^ moiety diffuses via a Grothuss proton‐hopping mechanism and is observed to be located ≈4.5 Å away from the Co atom in the transition state trajectory (*Q* = 0.26 Å) and more than 10 Å in the final state trajectory (*Q* = 1.4 Å). Earlier computational work, that employed an implicit solvent model reports a somewhat similar barrier of 17.1 kcal mol^−1^ but a substantially lower reaction energy of only 4.9 kcal mol^−1^. In this study, the carbonic acid was considered as the proton donor [4].

We also assessed, as an alternative metal–hydride formation step, the protonation of a Co‐coordinated N atom by a water molecule. Free, nonconstrained, DFT‐MD simulations with a proton initially close to Co–N site showed the H spontaneously moving to Co to form Co–H instead of N–H. This suggests that the formation of a N–H type catalyst is thermodynamically unfavorable.

#### 3.2.2 H–H Formation

Before evaluating the free‐energy profile of the hydrogen‐releasing step, we assessed the structure before and after a second electron reduction by free (nonconstraint) DFT‐MD simulations. These simulations showed that before reduction the Co─H bond average distance is around 1.47 Å. After reduction, that is, the initial state of H–H formation, this average bond distance is 1.43 or 1.57 Å, for the doublet and quartet spin state, respectively. This difference indicates a relatively weaker bonded, and, therefore more active Co─H, in a high spin state.

**FIGURE 5:**
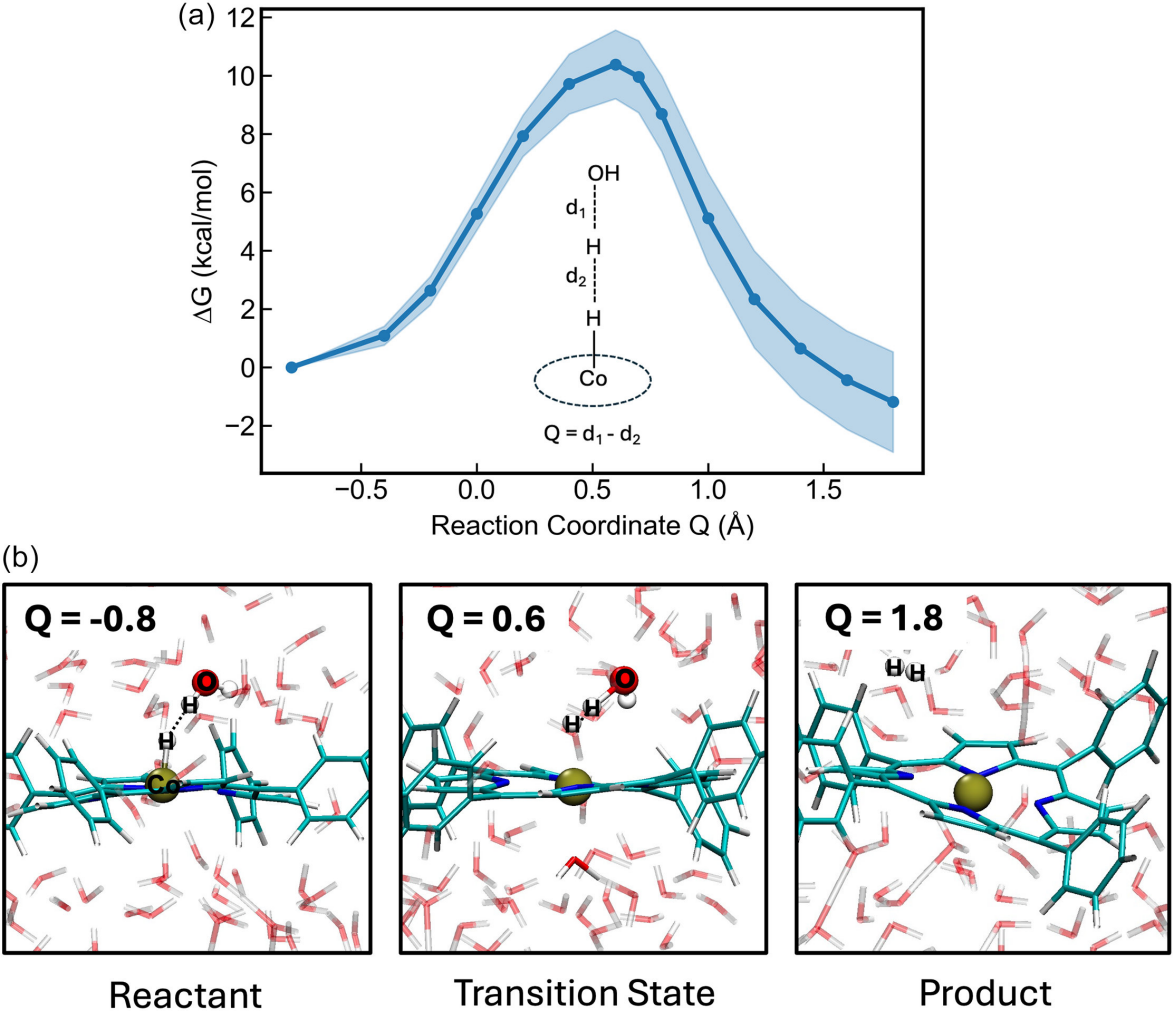
(a) Hydrogen formation by proton transfer from a solvent to the cobalt hydride, obtained from DFT‐MD simulation. The reaction coordinate (*Q*) is specified in the inset of free energy profile with error bar and (b) representative configurations near the reactant(*Q* = −0.8 Å), transition(*Q* = 0.6 Å) and product state (*Q* = 1.6 Å) are shown.

Assuming a quartet spin state, we determined the free energy profile for H_2_ production from [L–Co–H]^−^ with explicit aqueous solvent. The employed reaction coordinate *Q* involves a proton transfer from a solvent water molecule to the cobalt‐bound H, as indicated in the inset of Figure 5a. The value of *Q* was varied from −1.1 to 1.8 Å. The calculated free‐energy profile (Figure 5a) shows a transition‐state barrier of 10.6 kcal mol^−1^, and an exergonic reaction energy of −1.0 kcal mol^−1^. The representative snapshots shown in Figure 5a–c indicate how in the reactant state the [L–Co–H] moiety has relative strong hydrogen bond with a solvent molecule, which is indicative for the observed somewhat weaker Co─H bond. The product state trajectory where *Q *= 1.8 Å, H_2_ has been released from the Co center and moved into bulk solution, also illustrated in the Figure 5b snapshot.

Comparing the computed reaction barrier for the H─H bond formation (≈10 kcal mol^−1^) to that of the metal‐hydride formation step (≈22 kcal mol^−1^), demonstrates the latter can be considered the rate determining step for the HER.

In the broader context, with the RDS of the CO_2_RR having a computed barrier of ≈13 kcal mol^−1^ for the C─O bond breaking step, it appears from the model calculations presented in this paper that (kinetically) the CO_2_RR pathway is substantially more favorable than the HER pathway.

## 4 Conclusion

In summary, we performed DFT‐based molecular dynamics simulations incorporating explicit water to investigate the selectivity between electrochemical CO_2_‐to‐CO reduction and the competing HER in CoTPP catalyzed system. For the RDS of CO_2_RR, that is, C─O bond breaking, we find a barrier of around 13 kcal mol^−1^ while the RDS in HER, that is, cobalt hydride formation, is significantly higher (≈22 kcal mol^−1^). This suggests that the cobalt porphyrin molecule exhibits catalytic selectivity for CO_2_RR to CO rather than hydrogen generation.

The effect of the presence of a potassium cation was studied for key steps of the CO_2_RR. Here we considered two settings, adding an extra ion or replacing a water proton by an ion. Only moderate effects were observed that could originate from just a limited change in cation solvation energy for the CO_2_ binding step and a distant (second solvation shell) cation presence in the C─O bond breaking stage. In our view, this work may provide a proper basis for future (computational) electrocatalysis studies and system design in aqueous systems. On the theoretical and computational side, extending to models that mimic the experimental relevant conditions of imposed potential and/or pH would be important advancements, along with the (easy‐to‐be‐implemented) effect of cation concentration. The insights obtained with the current work also serve as a good starting point for elucidating the effect of other cations on CO_2_RR. On the long term, the findings may provide new directions for the rational (computational) design of molecular CO_2_RR catalysts in an aqueous environment, and thereby contributing to realizing more efficient renewable energy conversion systems.

## Funding

This work was supported by the China Scholarship Council (202106310031).

## Conflicts of Interest

The authors declare no conflicts of interest.

## Supporting information

Supplementary Material

## Data Availability

The data that support the findings of this study are available from the corresponding author upon reasonable request.
