# Supplementary material for: Elucidating the Role of Aqueous Solvent and Cations in Selectivity of Cobalt–Porphyrin Catalyzed CO2 Reduction Over Hydrogen Evolution
Source: Chemphyschem. 2026 Aug 12;27(15):e202500727. doi: 10.1002/cphc.202500727 (PMC13468187; doi:10.1002/cphc.202500727)
Supplement: Supplementary file 1 [file CPHC-27-e202500727-s001.pdf]

Supporting Information

Elucidating the Role of Aqueous Solvent and Cations in Selectivity of Cobalt–Porphyrin Catalyzed CO_2 Reduction Over Hydrogen Evolution

Lingshu Zhuo and Evert Jan Meijer*

Van 't Hoff Institute for Molecular Sciences, University of Amsterdam, Amsterdam The Netherlands

E-mail: e.j.meijer@uva.nl

Constraint Force Profiles

The calculated constraint force profiles used to determine the free energy profiles along selected reaction steps, including CO_2 binding, C-O breaking in CO_2RR with or without cation; Co-H formation, H-H formation in HER are provided below. The corresponding illustration of reaction coordinate Q is shown in the inset.

CO_2RR: C-O breaking

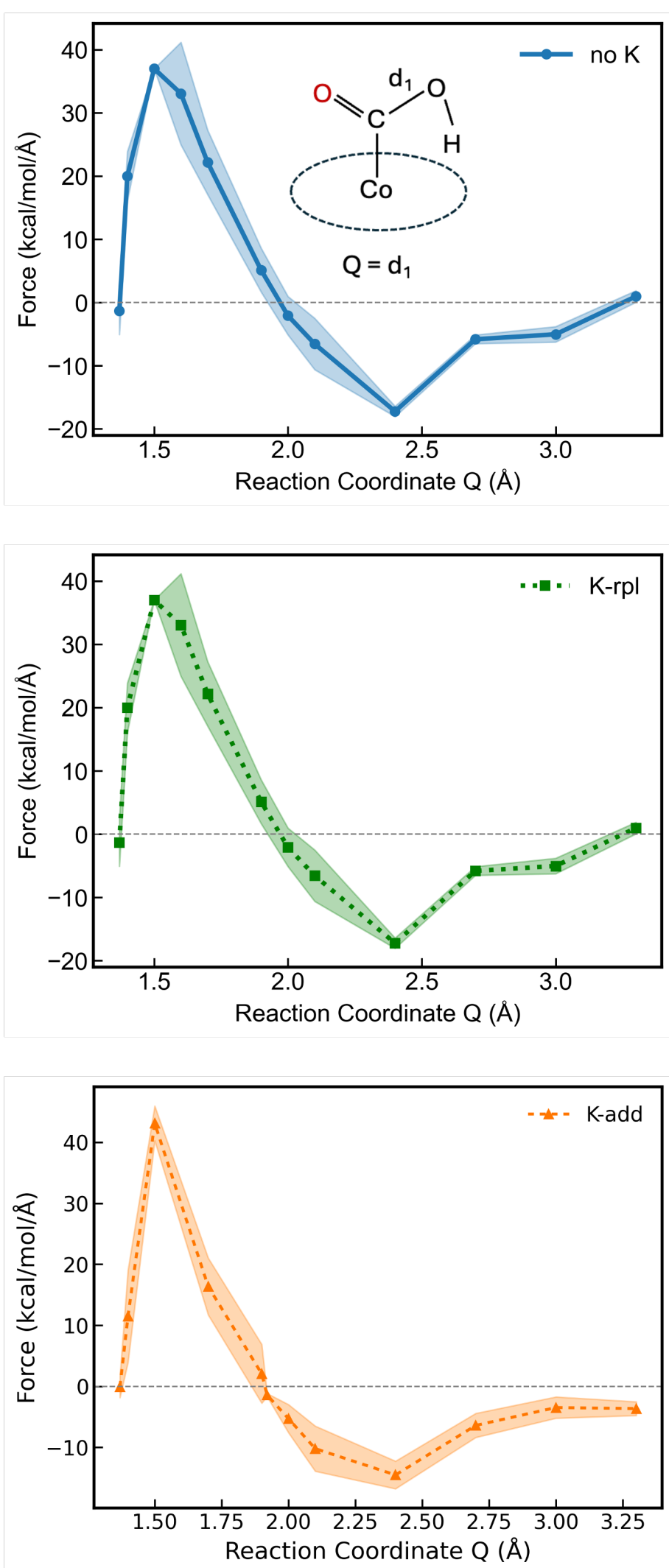

CO_2 binding: No K^+ ion

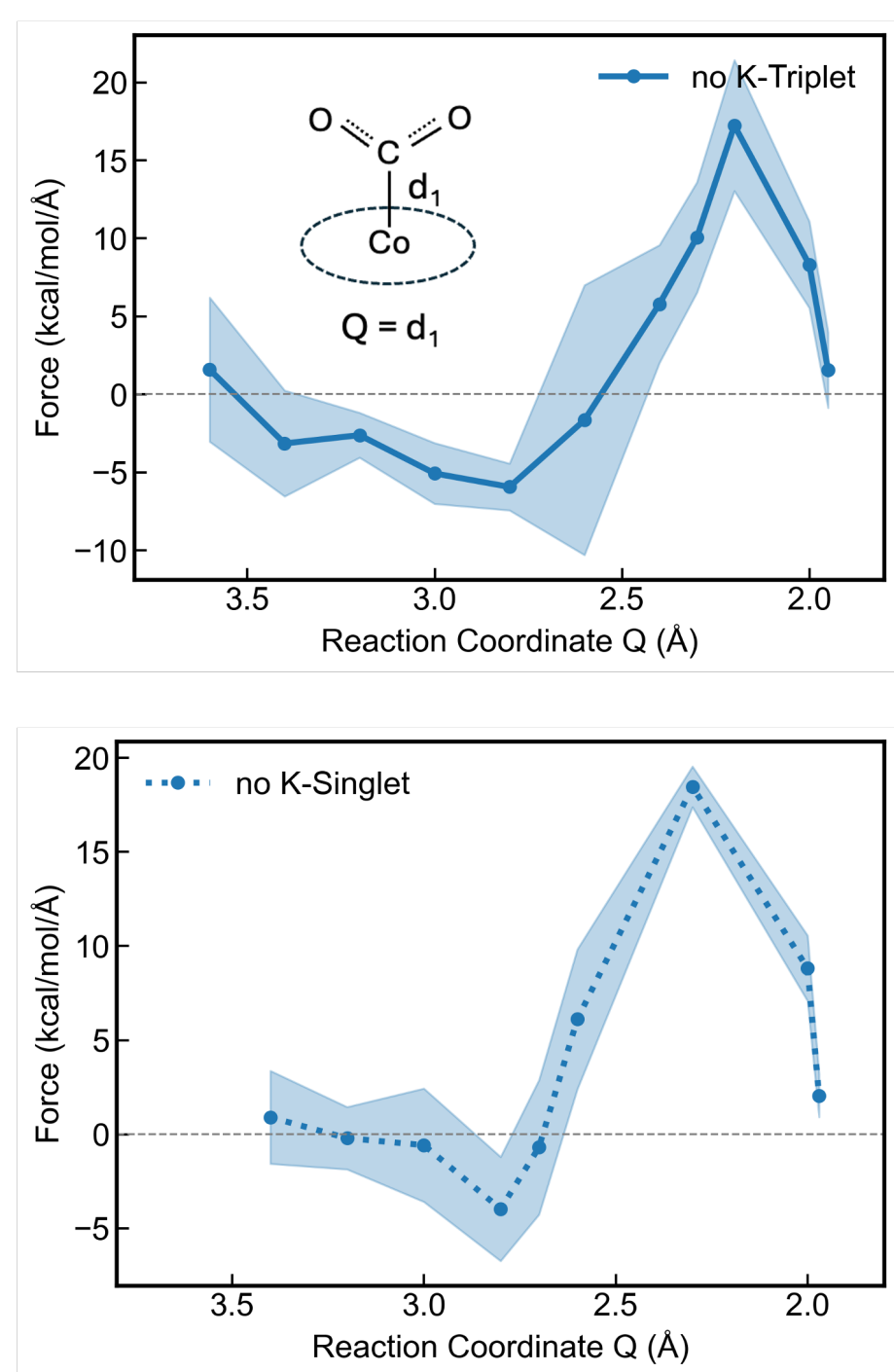

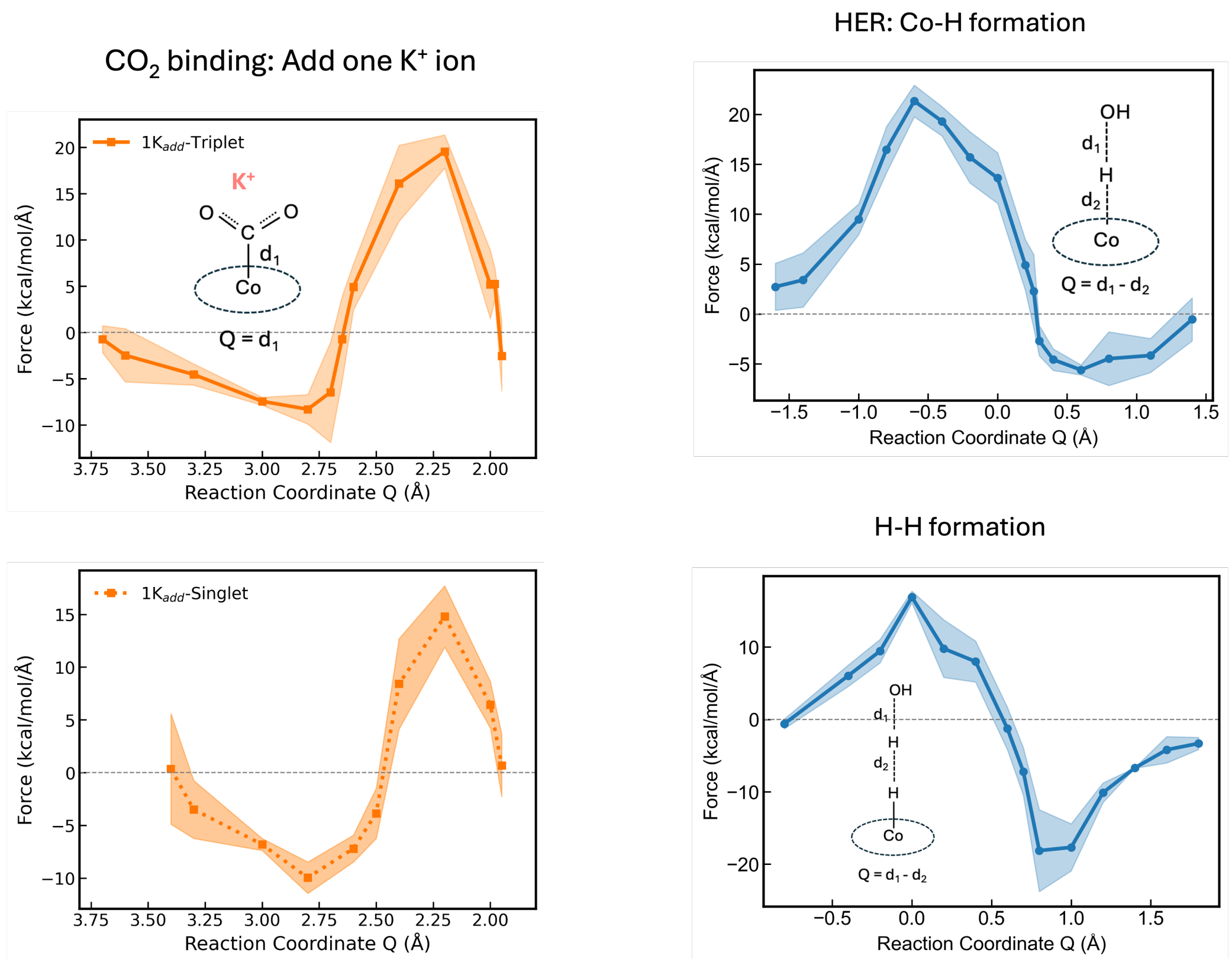
CO2 binding: Add one K+ ion
1K_add-Triplet
K+
O
C
O
d1
Co
Q = d1
Force (kcal/mol/Å)
Reaction Coordinate Q (Å)
1K_add-Singlet
Force (kcal/mol/Å)
Reaction Coordinate Q (Å)
HER: Co-H formation
OH
d1
H
d2
Co
Q = d1 - d2
Force (kcal/mol/Å)
Reaction Coordinate Q (Å)
H-H formation
OH
d1
H
d2
H
Co
Q = d1 - d2
Force (kcal/mol/Å)
Reaction Coordinate Q (Å)
